# Sensitivity to length and angle information develops early in PPA, but late in OPA

**DOI:** 10.1016/j.dcn.2026.101800

**Published:** 2026-08-15

**Authors:** Rebecca J. Rennert, Daniel D. Dilks

**Affiliations:** Emory University, United States

**Keywords:** Pediatric fMRI, Parahippocampal place area, Occipital place area, Scene processing, Development

## Abstract

Behavioral evidence suggests that visually-guided navigation develops later than scene categorization, yet how this dissociation is reflected in cortical organization remains unknown. In adults, it has been hypothesized that these abilities are supported by the occipital place area (OPA) and the parahippocampal place area (PPA), respectively. Here we tested whether OPA develops later than PPA using functional magnetic resonance imaging (fMRI) adaptation in children aged 5–8 years. Specifically, we measured neural sensitivity to length and angle information – features represented in both regions in adulthood – allowing a direct comparison of the developmental emergence of these representations. PPA exhibited robust sensitivity already at age 5, consistent with the earlier development of scene categorization. In contrast, OPA showed no reliable sensitivity at age 5, with robust sensitivity developing only between ages 5 and 8, consistent with later development of visually-guided navigation. Together, these findings reveal a developmental dissociation within scene-selective cortex: sensitivity to shared scene features emerges earlier in PPA than OPA, mirroring the distinct developmental trajectories of scene categorization and visually-guided navigation.

## Highlights

• Navigation within the immediately visible environment develops later than recognizing a kind of place.
• In adults, OPA supports such visually-guided navigation abilities and PPA supports such scene categorization abilities.
• We tested if OPA develops later than PPA by testing when regions are sensitive to geometry, using fMRI adaptation.
• Geometric representations emerge earlier and remain stable in PPA, whereas they develop gradually in OPA across childhood.

## 1 Introduction

A recent behavioral study found that visually-guided navigation (i.e., our ability to navigate within the immediately visible environment, avoiding boundaries and obstacles) develops years later than scene categorization (i.e., our ability to recognize a place as a particular kind of place) (Kamps et al., 2023). In this study, typically-developing children between the ages of 4 and 9 years performed two tasks (i.e., a scene categorization task and a visually-guided navigation task) on the exact same scene stimuli. Performance on the visually-guided navigation task reached adultlike performance around 8 years of age, but reached maturity much earlier on the scene categorization task, around 5 years of age. These behavioral findings not only provide the first evidence for differential development between scene categorization and visually-guided navigation, but also suggest that a similar developmental dissociation may be present at the level of the brain.

Importantly, at least in adults, neural evidence indicates that scene categorization and visually-guided navigation are supported by distinct scene-selective (i.e., responding more to scene than object stimuli) cortical systems. For example, it has been proposed that the parahippocampal place area, or PPA (Epstein and Kanwisher, 1998), supports scene categorization (Dilks et al., 2011, Dillon et al., 2018, Jung et al., 2018, Jung and Walther, 2021, Persichetti and Dilks, 2016, 2018, 2018, 2019; Walther et al., 2009, 2011). By contrast, the occipital place area, or OPA (Dilks et al., 2013), has been hypothesized to support visually-guided navigation (Bonner and Epstein, 2017, Jones et al., 2023, Julian et al., 2016, Kamps et al., 2016, Kamps et al., 2016, Kamps et al., 2025; J. Park and Park, 2020; Persichetti and Dilks, 2018). While the functional roles of PPA and OPA are relatively well-established in adulthood, much less is known about how these cortical regions develop. Paralleling the behavioral development of these systems, does OPA develop later than PPA?

Although the development of PPA and OPA have never been directly compared, a few pediatric neuroimaging studies have studied their development independently. More specifically, only 8-year-old OPA was sensitive to first-person perspective motion information (i.e., mimicking the visual experience of actually navigating through a place) (Jung et al., 2024, Kamps et al., 2020) and left and right information (Rennert and Dilks, 2025) – two pieces of information critical for visually-guided navigation and represented in OPA in adults (Dilks et al., 2011, Kamps et al., 2016) – whereas 5-year-old OPA was not at all sensitive to either kind of information. By contrast, another study found that 5-year-old PPA represented scene category information (Jung and Dilks, 2025), like adults (Persichetti and Dilks, 2019). Critically, however, none of these studies tested information shared by both PPA and OPA, and thus could not directly test whether OPA develops later than PPA. Furthermore, all these studies either compared two age groups (i.e., 5- and 8-year-olds) or only tested one age (i.e., 5-year-olds), and thus, the nature of this cortical development remains unclear. For example, does OPA develop gradually between ages 5 and 8, or does it develop abruptly somewhere in this age range? And is PPA fully developed by age 5, or is there more subtle development occurring after this age?

**Fig. 1:**
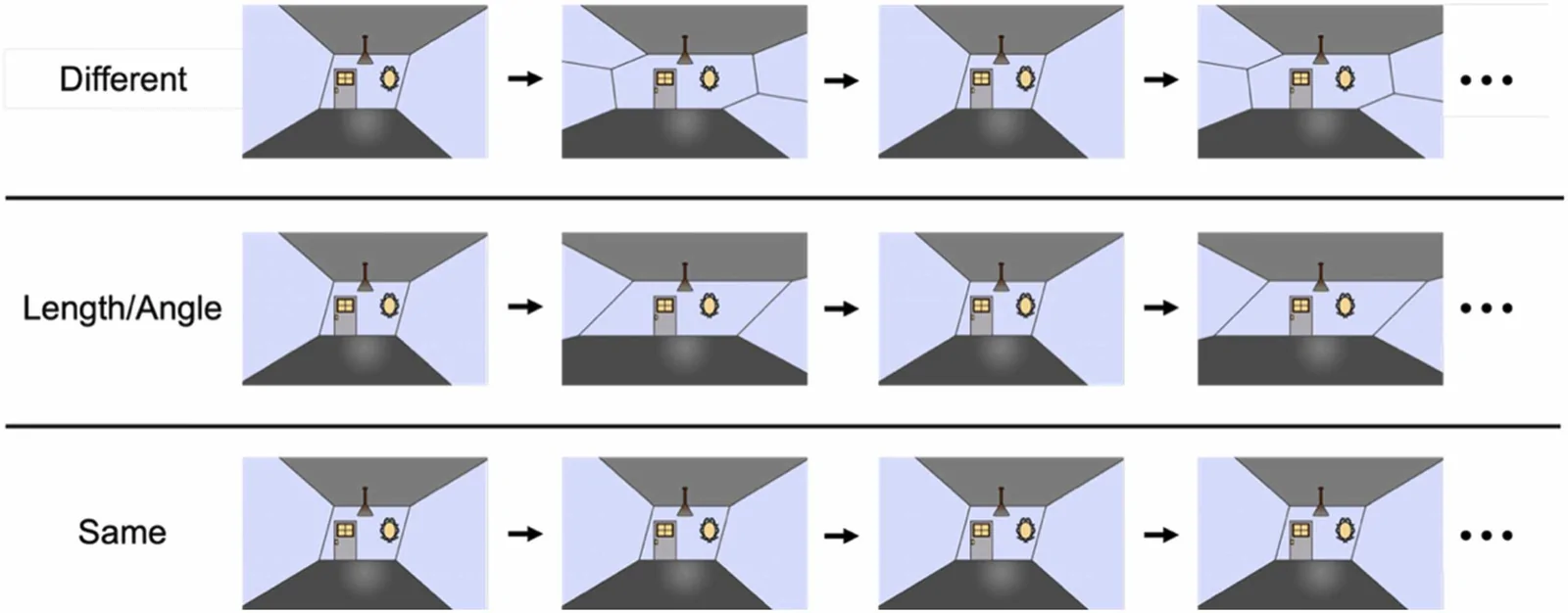
*Example stimuli used in the Different, Length/Angle, and Same conditions.* In the Different condition, participants viewed scene images that differed in both global shape – parallelogram or hexagon – and length and angle information (i.e., both different shape and different length and angle information). In the Length/Angle condition, participants viewed scene images that only differed by the relative lengths of surfaces and angles at which the surfaces meet (i.e., same shape, but different length and angle information). In the Same condition, participants viewed the same scene image (i.e., same shape and same length and angle information).

Here we overcome these two challenges by simultaneously testing the development of both PPA and OPA, within the same participants and over a continuous age range, using information that *both* regions represent in adulthood – that is, relative length and angle information that defines the geometric shape of the scene, which is critical for both scene categorization and visually-guided navigation, respectively (Dillon et al., 2018). More specifically, we used functional magnetic resonance imaging (fMRI) adaptation in children ages 5 through 8 years, and examined when in development these regions represent changes in length and angle information in scenes. Adapting an adult fMRI study (Dillon et al., 2018), children saw scene stimuli from three conditions: i) completely different scene images, differing in overall global shape (Different condition); ii) scene images that differ only by the relative lengths of surfaces and angles at which the surfaces meet (Length/Angle condition); or iii) the exact same scene image (Same condition) (Fig. 1). If a region is sensitive to the relative length and angle properties of scenes, then images presenting such changes will be treated as different images, and the neural activity will not show adaptation to these images. By contrast, if a region is insensitive to the relative length and angle properties of scenes, these images presenting such changes will be treated as the same image, and the neural activity will show adaption. Consistent with the hypothesis that OPA develops later than PPA, we predict that OPA will show sensitivity to length/angle information later in development than PPA, mirroring the late development of visually-guided navigation relative to scene categorization.

## 2 Methods

### 2.1 Participants

Fifty-six participants between 5 and 8 years of age participated in this study. Specifically, this sample included 16 5-year-olds (5 female, mean age = 67 months, range = 62–71 months), 16 6-year-olds (4 female, mean age = 78 months, range = 72–83 months), 12 7-year-olds (6 female, mean age = 87 months, range = 89–95 months), and 12 8-year-olds (6 female, mean age = 107 months, range = 102–109 months). Nine participants were excluded from analysis for excessive movement (3 5-year-olds, 4 6-year-olds, 1 8-year-old) or not paying attention (1 5-year-old), and thus 47 children (12 5-year-olds, 12 6-year-olds, 12 7-year-olds, and 11 8-year-olds) were included in the final analysis. Participants were recruited from the Emory University Child Study Center, a database of families interested in participating in research at Emory. All parents or legal guardians provided informed consent and all children over 5 years old provided informed assent. All participants were financially compensated for their participation, and all aspects of study procedures were approved under Emory University IRB. Finally, all participants had normal or corrected-to-normal vision and no history of neurological or psychiatric conditions.

Sample size for this cross-sectional design was determined based on prior developmental research using similar paradigms (Jung et al., 2024, Kamps et al., 2020, Rennert and Dilks, 2025). Although we did not conduct a power analysis a priori, we conducted sensitivity analysis for both categorical and continuous models of age (see *Data Analysis*), using a Type I error rate of .05 and target power of .80 (Faul et al., 2007). First, an ANOVA-based sensitivity analysis (treating age as a four-level factor) indicated that the study was powered to detect large between-group effects (f^2^ =. 50, corresponding ƞ_p_^2^ >.20). Next, a linear regression sensitivity analysis (treating age as a continuous predictor) indicated adequate power to detect moderate effects (f^2^ = 0.17, corresponding R^2^ >.15). While the sample size limits sensitivity to small effects, this is offset by design strengths including repeated-measures data across multiple runs per child, within-subject ROI sampling, and rigorous data quality controls. Finally, note that the experimental design itself also contains a built-in control, as adaptation (i.e., greater response to Different than Same conditions) is expected in all scene-selective regions in all participants (see *Experimental Design* and *Data Analysis*).

### 2.2 Experimental design

We used a region of interest (ROI) approach in which we used one set of runs (Localizer runs) to localize and define scene-selective regions. Then, we used a second set of runs (Experimental runs) to investigate the responses of these ROIs to changes in length and angle information in scenes. This ROI approach was facilitated by a group-constrained, subject-specific (GSS) method, described further in *Data Analysis*.

For the Localizer runs, participants viewed blocks of videos from two different conditions (i.e., “scenes” and “objects”). The scene stimuli consisted of 32 3 s video clips from a first-person perspective walking through a scene (Kamps, Lall, et al., 2016) and the object stimuli consisted of 32 3 s video clips of everyday objects moving dynamically (Pitcher et al., 2011). Each stimulus video subtended 12 × 9 ° visual angle. Each Localizer run was 180 s long and contained 3 blocks of each condition. The order of conditions was pseudorandomized across participants. Each block contained 6 3 s videos with an interstimulus interval (ISI) of 500 ms. Four 12 s fixation blocks were interspersed throughout the run: one at the beginning, and again after every two condition blocks. Participants were not instructed to do a formal task, but were told to pay attention to the videos.

For the Experimental runs, participants viewed blocks of scene images from three different conditions (i.e., “Different,” “Length/Angle," and “Same”). The stimuli were previously used in adult fMRI study investigating the representation of length and angle information across scene-selective cortex (Dillon et al., 2018). More specifically, the stimuli consisted of 6 images of computer-generated scenes of indoor rooms. The back wall of each room was a unique shape (i.e., parallelogram or hexagon), with a different side-length ratio (i.e., 1:1.33, 1:1.66, 1:1.99) and a different pair of supplementary angles (i.e., 105°/75°, 120°/60°, 135°/45°) (Fig. 1). The distance from the front end of the picture and the back wall of the room was held constant across stimuli. Thus, each stimulus depicted a room with a unique combination of length and angle information, where “length” refers to the length of the back wall and “angle” refers to the angle where the back wall joins the side walls. Each stimulus subtended 12 × 9 ° visual angle. Each Experimental run was 224 s long and contained 3 blocks of each condition. The order of conditions was pseudorandomized across participants. Each condition block contained 12 images displayed for 800 ms with an ISI of 500 ms. In the Different condition, participants viewed one stimulus followed by another stimulus differing in both shape and length/angle, and this pairing repeated for the rest of the block; in the Length/Angle condition, participants viewed one stimulus followed by another stimulus differing *only* in length/angle, and this pairing repeated for the rest of the block; and in the Same condition, participants viewed one repeated stimulus. Ten 8 s fixation blocks were interspersed throughout the run: one at the beginning, and again after every block. Participants were not instructed to do a formal task, but were told to pay attention to each stimulus. Note although there was no formal task, we implemented multiple measures to ensure participants were paying attention (see *2.4 Data Analysis*), including using the expected adaptation effect (i.e., greater response to Different than Same condition, regardless of region or age) and general activation in primary visual cortex (V1).

During each scanning session, we first took a high-resolution anatomical scan while the participant watched a movie of their choice. Then, we collected fMRI data while participants viewed 2 Localizer runs and 4 Experimental runs.

### 2.3 fMRI scanning

All scanning was performed on a 3 T Siemens Trio scanner in the Facility for Education and Research in Neuroscience at Emory University. The functional images were collected using a 32-channel head matrix coil and a gradient-echo single-shot echoplanar imaging sequence (28 slices, TR = 2 s, TE= 30 ms, voxel size = 1.5 × 1.5 × 2.5 mm, and a 0.5 interslice gap). For all scans, slices were acquired parallel to the anterior-posterior commissure, covering all the occipital and temporal lobes, as well as the lower portion of the parietal lobe. Additionally, whole-brain, high-resolution anatomical images were acquired for each participant for registration and anatomical localization.

### 2.4 Data analysis

fMRI data was preprocessed using AFNI (Cox, 1996) (version 22.0.02). Data from the Experimental runs were registered to a T1-weighted reference using align_epi_anat.py (AFNI) and corrected for head-motion using 3dvolreg (AFNI). Before motion correction, volumes with large artifacts were corrected via interpolation between the nearest nonaffected volumes to reduce abrupt signal changes caused by head motion (3dDespike, AFNI). Spatial smoothing was applied with a Gaussian kernel with a 6 mm full width at half maximum (FWHM) (3dmerge, AFNI). High pass temporal filtering was performed using 3dTproject (AFNI) to remove any frequencies above 0.2 Hz. Head-motion parameters with respect to the BOLD reference were estimated before any spatial or temporal smoothing. Then, 3dDeconvolve (AFNI) was used to model the data using a General Linear Model (GLM). Specifically, the onset and duration of each condition block (i.e., Different, Length/Angle, Same, Fixation) was convolved with a canonical hemodynamic response function (HRF). Six estimated motion parameters (three translations: x, y, z; and three rotations: roll, pitch, yaw) were also included in the model as nuisance regressors. Finally, any datapoints with motion displacement greater than 1.5 mm were censored from the regression. Together, this model yielded three beta values (percent signal change, or PSC, for each of the three experimental conditions relative to baseline fixation). Adaptation was thus measured in the following ways. The adaptation effect to scenes was calculated as the difference between the Different and Same conditions. Adaptation to length and angle information (i.e., length/angle sensitivity) was measured as the difference between the Length/Angle and Same conditions.

To ensure we only analyzed the highest quality data in our sample, we only analyzed runs where the average absolute frame-to-frame displacement was less than 1.5 mm (i.e., the approximate size of one voxel), and where activation could be detected in V1 (Z > 2.3, corrected cluster significance threshold of p = .05). As previously described, these criteria resulted in the exclusion of 9 participants for excessive motion across all runs and 1 participant for insufficient V1 activity across all runs (i.e., entire participants were excluded only if all runs exceeded these cutoffs). As a result of these procedures, the final group of participants were matched on head motion (i.e., average framewise displacement for all usable runs) (no significant effect of Age on motion; F_(3,43)_ = 0.68, p = .57, ƞ_p_^2^ = .05), temporal signal to noise ratio (tSNR) in all ROIs (no significant effect of Age on tSNR for any region; PPA: F_(3,43)_ = 1.43, p = .25, ƞ_p_^2^ = .09; OPA: F_(3,43)_ = 1.22, p = .31, ƞ_p_^2^ = .08), and V1 activation (i.e., the average response in V1 across all conditions) (no significant effect of Age on V1 activation; F_(3,43)_ = 1.87, p = .15, ƞ_p_^2^ = .11). tSNR was calculated after smoothing, removing motion and baseline trends, and detrending. The number of usable runs from each age group was also comparable (no significant effect of Age on number of useable runs; F_(3,43)_ = 0.54, p = 66, ƞ_p_^2^ = .04), resulting in an average of 3.3 usable runs per participant (7 participants had 2 usable runs, 19 participants had 3 usable runs, and 21 participants had 4 usable runs).

Given that hand-defining regions-of-interest (ROIs) can be ambiguous, particularly at earlier stages of development, ROIs were facilitated by a Group-Constrained Subject Specific (GSS) method that circumvents these challenges (Fedorenko et al., 2010). First, for each participant, we identified a search space for each ROI using previously published probabilistic atlases that predict the location in which each ROI is likely to fall given the typical distribution found in a large sample of adults. These atlases are published in standard MNI space and were aligned to each individual subject’s native space using 3dNwarpApply and 3drefit (AFNI). Search spaces for scene-selective regions (OPA, PPA, and the retrosplenial complex, or RSC – a third scene region that does *not* represent length/angle information; Dillon et al., 2018) were derived from Jung et al. (2026), while search spaces for V1 were derived from Wang et al. (2015). Notably, these scene-selective search spaces are appropriate for both adult and pediatric populations, as tested here (Jung et al., 2026). Second, for each search space in each participant, voxels were ranked using the data from the localizer runs, based on parameter estimates for the contrasts of scenes > object videos (for the scene-selective regions), or all conditions > fixation (for V1). Based on these rankings, the top 10% of the voxels were then selected as the subject-specific ROI. Third, responses to each condition in each ROI and participant were measured using the remaining, independent experimental runs. Percent signal change was calculated as the average response to each condition relative to baseline fixation (averaged over blocks). For each ROI, GSS analysis was conducted separately in each hemisphere. None of the ROIs had a significant hemisphere x condition interaction (all F’s < 2.33, all p’s > .10, all ƞ_p_^2^ <.18), and thus the responses from the ROIs in each hemisphere were subsequently averaged together.

To directly test the hypothesis that OPA represents length/angle information in scenes later than PPA, we first used repeated-measures ANOVAs to compare responses in each ROI to each condition within each age group (binned by year). We also used the mgcv package in R (Wood, 2000) to create generalized additive models (GAMs), which investigate how responses to each condition (i.e., PSC) changed continuously over our participant age range (i.e., month age) without assuming a linear relationship between PSC and age. Age was modeled using a penalized thin plate regression spline, with smoothing parameters estimated via restricted maximum likelihood (i.e., REML) and assuming a Gaussian distribution.

## 3 Results

### 3.1 OPA represents length/angle information in scenes later in development than PPA

**Fig. 2:**
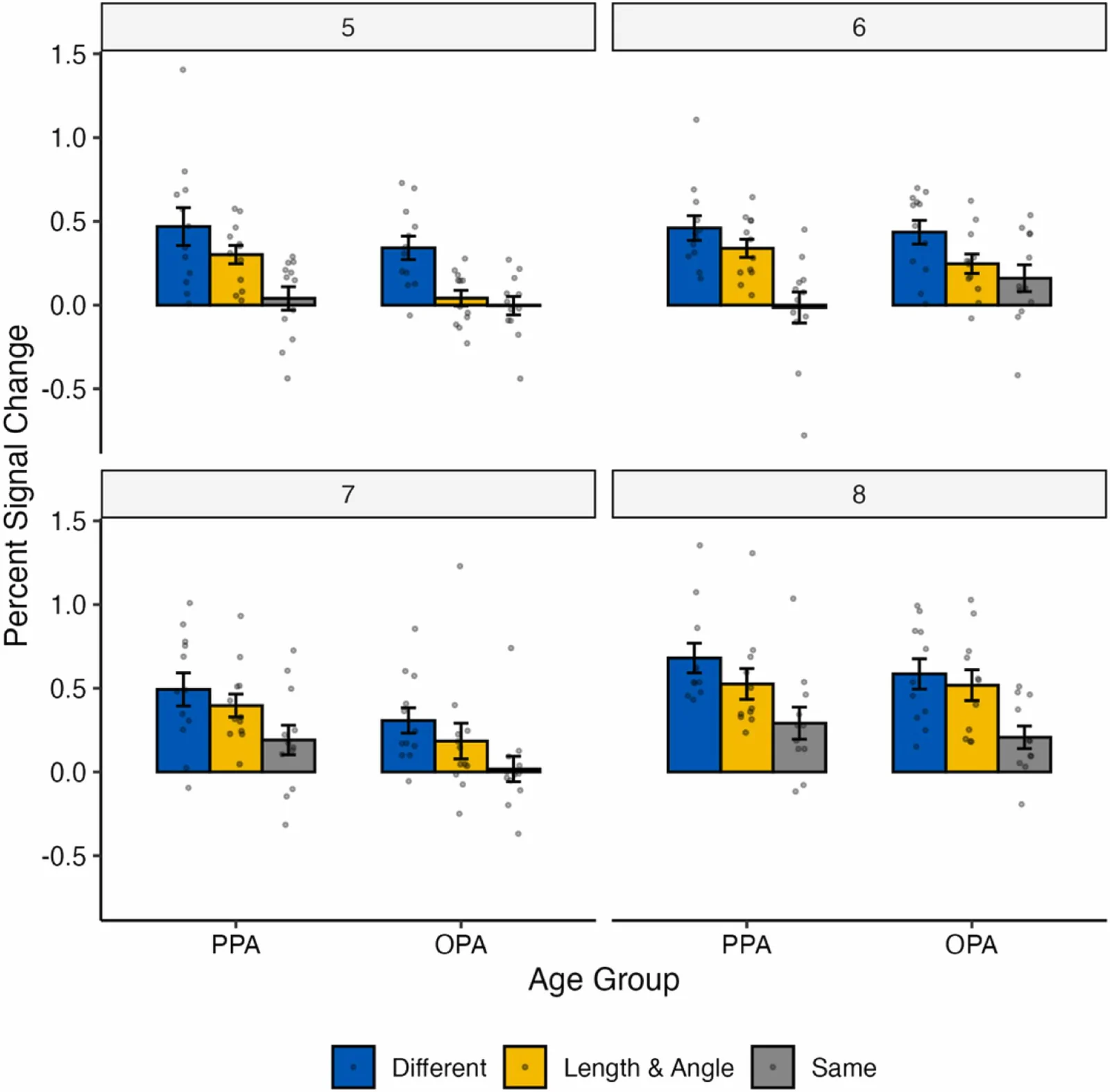
*OPA represents length/angle information in scenes later in development than PPA*. Percent signal change (y-axis) to each experimental condition (Different: blue, Length/Angle: yellow, Same: grey) relative to a baseline fixation for PPA (left) and OPA (right) for 5-, 6-, 7-, and 8-year-old participants. Individual dots represent data from individual participants. Bars represent the mean response to a particular condition for a particular age group (x-axis), and error bars represent SEM.

If OPA develops later than PPA, then PPA should represent length/angle information in scenes earlier in development (e.g., in 5-year-olds) than OPA (e.g., in 7 or 8-year-olds). To test this hypothesis, we first tested how both regions responded to the experimental conditions within each age group (Fig. 2).

First, in 5-year-olds, a 2(ROI: PPA, OPA) x 3(Condition: Different, Length/Angle, Same) repeated-measures ANOVA revealed a significant ROI x Condition interaction (F_(2, 22)_ = 4.32, p < .05, ƞ_p_^2^ = .28), as well as a significant effect of Condition (F_(1, 11)_ = 26.66, p < .001, ƞ_p_^2^ = .71). Interaction contrasts revealed that while both PPA and OPA demonstrate the expected adaptation effect in 5-year-olds (Different > Same condition; all t’s > 4.35, all p’s < .01), only PPA demonstrates sensitivity to length/angle information (Length/Angle > Same condition, t_(11)_ = 5.63, p < .001), while OPA does not (t_(11)_ = 1.63, p = .27). A Bayesian paired-samples *t*-test was then conducted to evaluate evidence for the absence of a difference between the Length/Angle and Same conditions in 5-year-old OPA. This analysis revealed BF_10_ = 0.82, which provides anecdotal evidence for the null hypothesis (Jeffreys, 1961).

Similarly, in 6-year-olds, a 2(ROI: PPA, OPA) x 3(Condition: Different, Length/Angle, Same) repeated-measures ANOVA revealed a significant ROI x Condition interaction (F_(2, 22)_ = 4.19, p < .05, ƞ_p_^2^ = .28), as well as a significant effect of Condition (F_(1, 11)_ = 25.57, p < .001, ƞ_p_^2^ = .72). Like the 5-year-olds, interaction contrasts revealed that both PPA and OPA demonstrate the expected adaptation effect (Different > Same condition; all t’s > 3.59, all p’s < .01). Additionally, also like the 5-year-olds, only PPA demonstrates sensitivity to length/angle information (Length/Angle > Same condition, t_(11)_ = 4.63, p < .01), but OPA does not (t_(11)_ = 1.32, p = .42). To further explore the absence of a difference between the Length/Angle and Same conditions in the 6-year-old OPA, we again conducted a Bayesian paired-sample *t*-test, which revealed anecdotal evidence for the null hypothesis (BF_10_ = 0.58).

However, in 7-year-olds, a 2(ROI: PPA, OPA) x 3(Condition: Different, Length/Angle, Same) repeated-measures ANOVA did not reveal a significant ROI x Condition interaction (F_(2, 22)_ = 0.16, p = .86, ƞ_p_^2^ = .01), but did reveal a significant effect of Condition (F_(1, 11)_ = 15.60, p < .001, ƞ_p_^2^ = .59). Main effect contrasts (collapsing over ROIs) then revealed successful adaptation (Different > Same condition, t_(11)_ = 5.25, p < .001), as well as sensitivity to length/angle information (Length/Angle > Same condition, t_(11)_ = 3.32, p < .05). These results indicate that by age 7, both PPA and OPA are sensitive to length/angle information in scenes.

Finally, in 8-year-olds, a 2(ROI: PPA, OPA) x 3(Condition: Different, Length/Angle, Same) repeated-measures ANOVA also did not reveal a significant ROI x Condition interaction (F_(2, 20)_ = 0.90, p = .42, ƞ_p_^2^ = .08), but nevertheless did reveal a significant effect of Condition (F_(1, 10)_ = 27.94, p < .001, ƞ_p_^2^ = .74). Main effect contrasts (collapsing over ROIs) further revealed successful adaptation (Different > Same condition, t_(10)_ = 5.61, p < .001), as well as sensitivity to length/angle information (Length/Angle > Same condition, t_(10)_ = 5.27, p < .001). Thus, both PPA and OPA are sensitive to length/angle information in scenes in 8-year-olds.

Given that both regions demonstrated the expected adaptation effect in all age groups, we next asked how length/angle sensitivity, specifically, develops within each region. In PPA, a 4(Age: 5-, 6-, 7-, 8-year-olds) x 2(Condition: Length/Angle, Same) ANOVA did not reveal a significant Age x Condition interaction (F_(3,43)_ = .97, p = .42, ƞ_p_^2^ = .06), suggesting that PPA has a stable representation of length/angle information between ages 5 and 8 (i.e., no development of length/angle sensitivity within this age range). In OPA, however, a 4(Age: 5-, 6-, 7-, 8-year-olds) x 2(Condition: Length/Angle, Same) ANOVA revealed a significant Age x Condition interaction (F_(3,43)_ = 4.23, p < .01, ƞ_p_^2^ = .23), with greater sensitivity to length/angle information in 5- and 6-year-olds compared to 8-year-olds (Length/Angle > Same, main effect contrasts; 5 vs. 8: t_(43)_ = 3.32, p < .01; 6 vs. 8: t_(43)_ = 2.79, p < .01).

Finally, we directly compared the development of length/angle sensitivity in PPA and OPA. A 2(ROI: PPA, OPA) x 4(Age: 5-, 6-, 7-, 8-year-olds) x 2(Condition: Length/Angle, Same) ANOVA revealed a significant interaction (F_(3,43)_ = 4.57, p < .01, ƞ_p_^2^ = .24), driven by greater length/angle sensitivity in 8-year-old OPA relative to 5-year-old OPA (interaction contrast, t_(43)_ = 3.46, p < .05) and in 8-year-old PPA relative to 5-year-old OPA (interaction contrast, t_(43)_ = 3.88, p < .01). Together, these results demonstrate that sensitivity to length/angle information develops earlier in PPA (by age 5) than in OPA (emerging at the group-level, at least by age 8), confirming that OPA develops later than PPA and revealing a developmental lag in geometric representations within scene-selective cortex.

But could the responses in PPA or OPA simply reflect information inherited from low-level vision? In contrast to scene-selective cortex, early visual cortex (V1; see Methods) showed no evidence of adaptation, with no significant difference between the Different and Same conditions in any age group (main effect contrasts, all t’s < .92, all p’s > .64), nor differentiate between the three experimental conditions (i.e., no significant effect of Condition in any age group, all F’s < 2.16, all p’s > .14, all ƞ_p_^2^ <.16), likely because participants were not required to fixate (Rennert and Dilks, 2025) (Supplemental Figure 1). These results suggested the observed effects in scene-selective cortex cannot be explained by inheritance of low-level visual information from upstream processing.

Similarly, could the late development of length/angle information in OPA simply reflect a lack of scene selectivity (i.e., greater responses to scene than object stimuli)? We used the GSS method (see Methods) to compare responses in both PPA (for completeness) and OPA to scene and object stimuli from the localizer runs, using half of the runs to define these regions and the other half to test. In all age groups, PPA responded significantly more to scenes than objects (pairwise *t*-tests; 5: t_(6)_ = 6.74, p < .001, d = 2.55, 6: t_(8)_ = 7.66, p < .001, d = 2.55, 7: t_(8)_ = 17.81, p < .001, d = 2.60, 8: t_(5)_ = 18.6, p < .001, d = 7.61). Similarly, OPA also responded significantly more to scenes than objects (pairwise *t*-tests; 5: t_(6)_ = 3.71, p < .01, d = 1.40, 6: t_(8)_ = 4.45, p < .01, d = 1.48, 7: t_(8)_ = 2.43, p < .05, d = 0.81, 8: t_(5)_ = 3.12, p < .05, d = 1.27). Furthermore, neither PPA nor OPA showed a significant 4(Age: 5-, 6-, 7-, and 8-year-olds) x 2(Condition: Scenes, Objects) interaction (PPA: F_(3,27)_ = 0.29, p = .83, ƞ_p_^2^ = .03; OPA: F_(3,27)_ = 0.29, p = .83, ƞ_p_^2^ = .03), suggesting that scene selectivity does not develop across this age range (Supplemental Figure 2*)*.

For completeness, we also investigated how the retrosplenial complex (RSC; Maguire, 2001) – a third scene-selective region involved in map-based navigation – represents length/angle information in scenes between ages 5 through 8. Map-based navigation refers to the ability to navigate from a current location to some distant, out-of-sight location (Dilks et al., 2022), and does not require encoding of local geometric structure. Accordingly, RSC does not represent length/angle information in adults (Dillon et al., 2018) and therefore we predicted no such sensitivity in children. Across all age groups, a 3-way (Condition: Different, Length/Angle, Same) repeated-measures ANOVA revealed a significant effect of condition (all F’s > 4.67, all p’s < .05, all ƞ_p_^2^ >.30), with all age groups showing the expected adaptation effect (Different > Same condition; main effect contrasts, all t’s > 3.06, all p’s < .05) but no sensitivity to length/angle information (Length/Angle >Same condition; main effect contrast, all t’s > 1.67, all p’s > .26) (Supplemental Figure 3). These results reveal that, RSC does not represent changes in relative length/angle information between ages 5 and 8, like adults (Dillon et al., 2018), consistent with its role in map-based navigation.

Next, how does object-selective cortex – the lateral occipital complex (LOC) – respond to length and angle information, even in scenes? Interestingly, in adults, LOC is sensitive to length and angle changes in both objects *and* scenes (Dillon et al., 2018). While this study was not originally designed to test how this information develops within LOC, we nonetheless asked this question in an exploratory analysis (see Supplemental Materials). In brief, LOC does not represent length and angle changes in scenes in children ages 5–8 (Supplemental Figures 4 and 5), suggesting that this information may develop late (similar to other object properties, such as viewpoint invariance; Nishimura et al., 2015).

**Fig. 3:**
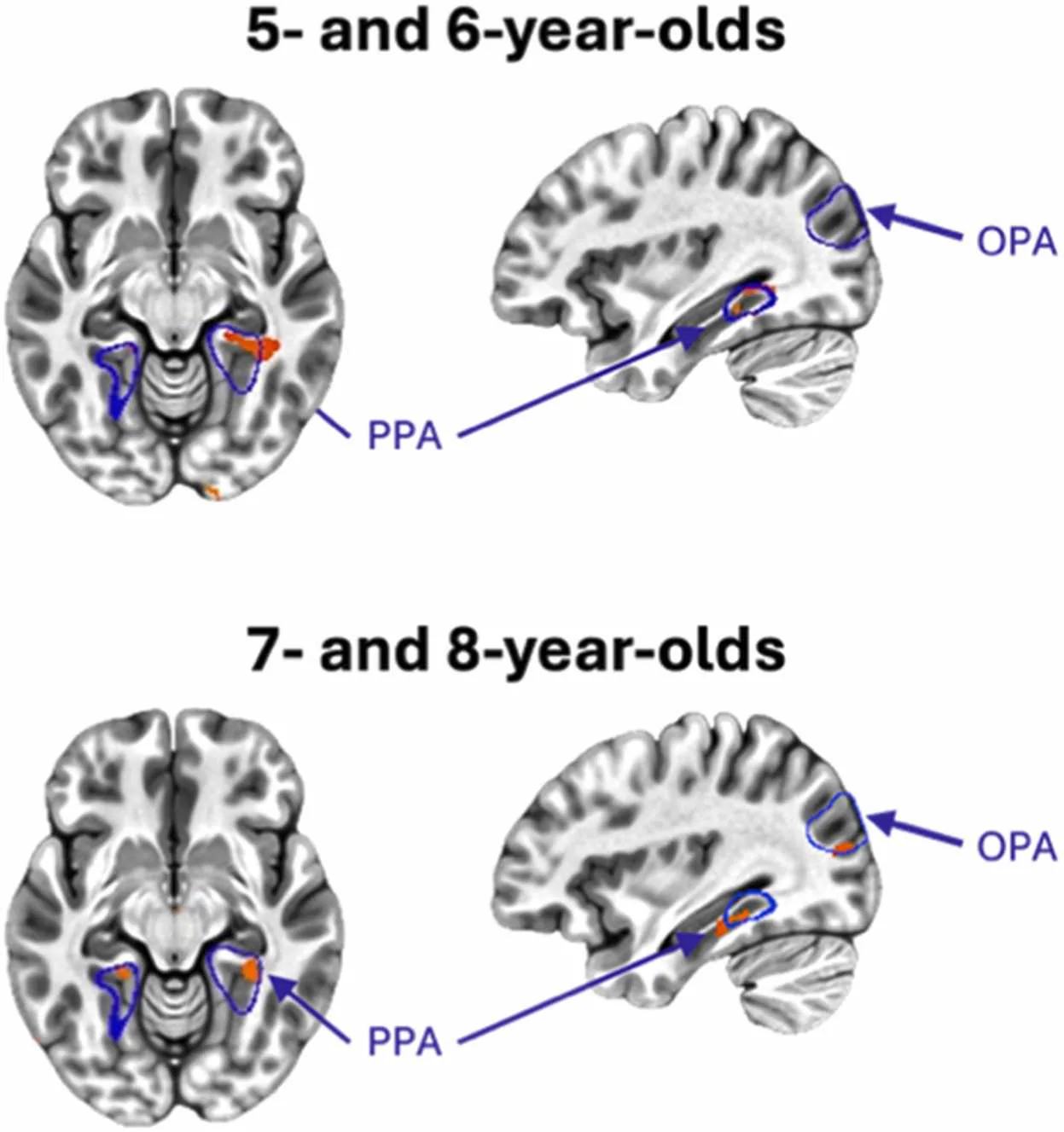
*Whole-brain analyses reveal regions representing length/angle information in scenes*. Whole-brain analyses for 5- and 6-year-olds (upper) and 7- and 8-year-olds (lower) show clusters (in red) with greater activation to the Length/Angle than Same conditions (thresholded at p < .01). Blue lines denote the location of PPA and OPA.

Are there other regions that may show sensitivity to length and angle information in scenes, outside of the ROIs already tested? To investigate this possibility, we ran a whole-brain analysis, combing data from the 5- and 6-year-olds and 7- and 8-year-olds to form “younger” and “older” groups, respectively. This analysis revealed a cluster within PPA in both the younger and older participants, as well as a cluster within OPA in the older participants (Fig. 3*)*. This analysis did not reveal sensitivity elsewhere. Thus, the whole-brain analysis is consistent with the ROI-based finding that OPA represents length and angle information in scenes later in development than PPA.

### 3.2 But what is the precise nature of length/angle development in OPA?

While our findings thus far reveal that length/angle representation in OPA changes between ages 5 and 8, the precise nature of this development remains unclear. One possibility is that sensitivity to length/angle information emerges abruptly in OPA, coming online suddenly somewhere between ages 5 and 8. Alternatively, this representation may emerge gradually, increasing in strength between ages 5 and 8. To distinguish between these accounts, we examined length/angle sensitivity in OPA (computed as the difference between Length/Angle and Same conditions) as a continuous function of age.

A GAM (see Methods) revealed a significant smooth term for age (F_(1.3,45)_ = 7.80, p < .01), confirming that length/angle sensitivity in OPA increases with age. Age explained 20.2% of the deviance (adjusted R^2^ =.18). The estimated degrees of freedom (EDF of 1.16) indicated an approximately linear relationship between age and length/angle sensitivity (EDF = 1 reflects linearity; Wood, 2017). Model predictions further indicated that length/angle sensitivity becomes significantly greater than zero at 71.5 months (just under 6 years). Notably, this earlier emergence was not fully captured by the prior categorical analyses, in which reliable effects were observed only in 7- and 8-year-olds, and group differences were primarily driven by contrasts with the oldest age group. Together, these results suggest that length/angle sensitivity in OPA emerge gradually, beginning around age 6, but only reaches robust group-level reliability later in development, around age 8, and thus highlighting the greater sensitivity of continuous developmental modeling to detect emerging neural representations.

For completeness, we applied the same GAM approach for PPA. The model revealed a nonsignificant smooth term for age (F_(1,45)_ = 0.46, p = .50) with an EDF of 1 and age explaining 1% of the deviance (adjusted R^2^ = −0.02). Model predictions further indicated that length/angle representation was already significantly different from zero at 62 months, corresponding to the age of the youngest age tested. These findings are consistent with earlier analyses, showing that sensitivity to length/angle information is already present in PPA by age 5, and remains stable across development from ages 5–8.

**Fig. 4:**
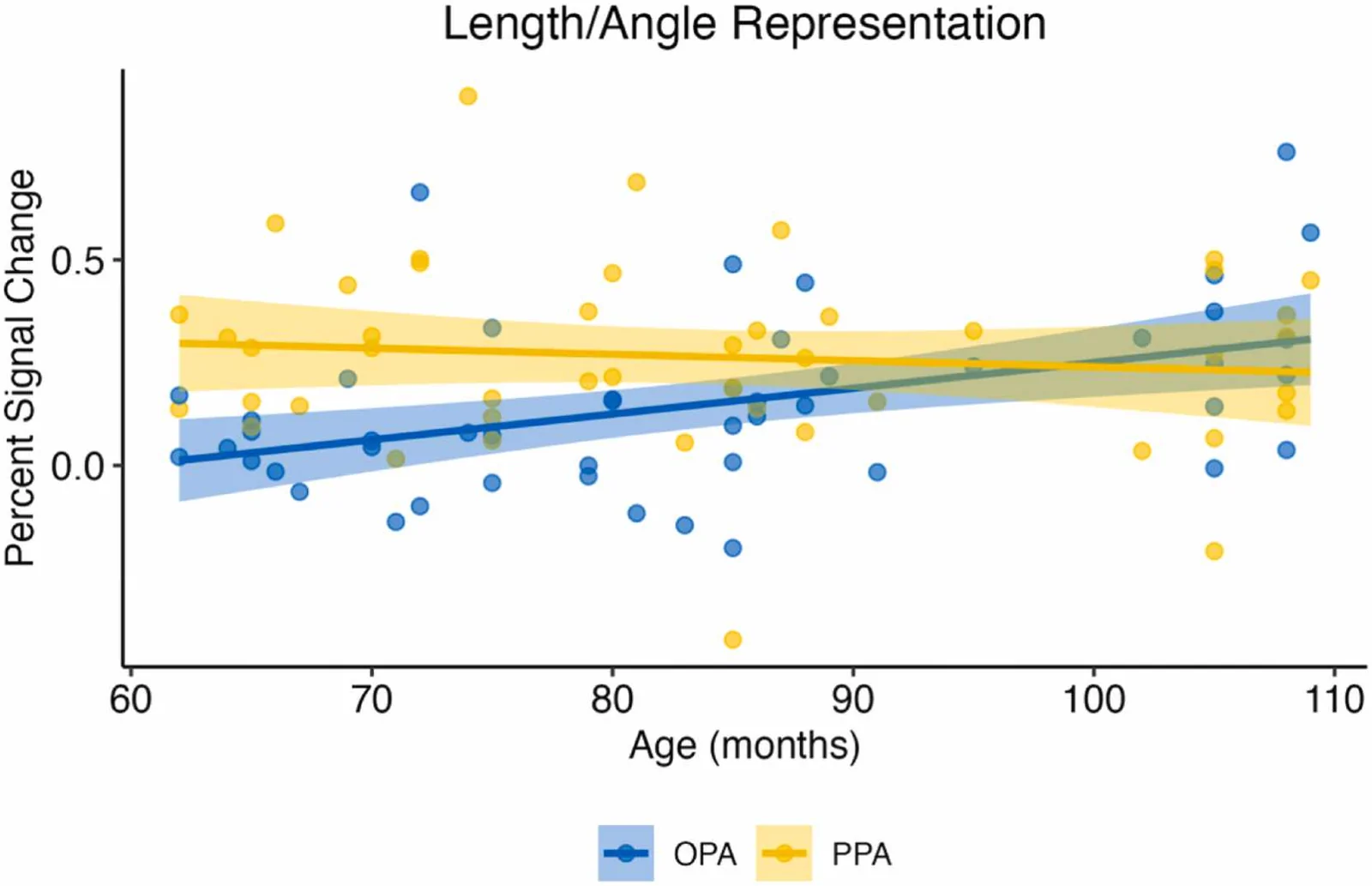
*Length/angle information in scenes is gradually represented in OPA between ages 5 through 8, yet is already represented in PPA by age 5.* Percent signal change (y-axis) to length/angle representation in OPA (blue) and PPA (yellow) between 5 through 8 years of age (x-axis, continuous in months). Individual dots represent individual length/angle representation (calculated as the difference in PSC between the Length/Angle and Same conditions). A linear model (solid lines) was fit to the data to describe the development of length/angle representation in each region (shaded regions represent a 95% CI).

Finally, we directly compared the developmental trajectories of length/angle sensitivity in OPA and PPA between ages 5 though 8 using a linear trend analysis (for model simplicity, as a linear function best described both trajectories; Fig. 4). This analysis revealed a significant interaction between region and age (F_(1,45)_ = 9.36, p < .01, ƞ_p_^2^ = .17), reflecting increasing length/angle sensitivity in OPA (b = 0.006, SE = 0.002, 95% CI [0.002, 0.010]) but no reliable change in PPA (b = −0.001, SE = 0.002, 95% CI [−0.006, 0.003]). Thus, length/angle representations emerge earlier and remain stable in PPA, whereas they develop gradually in OPA across childhood, confirming a dissociation in developmental timing between scene categorization and visually-guided navigation systems.

## 4 Discussion

The current study tested the hypothesis that OPA develops later than PPA by using fMRI adaptation in children ages 5 through 8 to examine when in development these cortical systems represent length and angle information. Consistent with this hypothesis, we found that OPA is sensitive to length/angle information later in development than PPA, with PPA already robustly sensitive to such information by age 5 while OPA gradually develops sensitivity to this information between the ages of 5 and 8, with robust group-level sensitivity only emerging by age 8. These findings mirror the distinct developmental trajectories of scene categorization and visually-guided navigation abilities.

Although the current study did not explicitly examine scene categorization abilities, theories of scene categorization suggest that such abilities depend on spatial layout representations (e.g., a beach has an “open” layout versus a hallway has a “closed” layout) (Oliva and Torralba, 2001, Oliva and Torralba, 2002), of which PPA (in adults) demonstrates sensitivity (Epstein and Kanwisher, 1998, Kamps et al., 2016, Kravitz et al., 2011; S. Park et al., 2011). Interestingly, our finding that PPA robustly represents length/angle information by age 5 is consistent with other behavioral studies showing that scene categorization abilities are well-developed by this age. For example, even infants can detect changes in scene category (Blesic et al., 2023, Duh and Wang, 2014). In one study, infants looked longer at stimuli where the “scene gist” (i.e., the scene category) was interrupted than when scene gist was preserved, suggesting that young children have may expectations about scene category (Duh and Wang, 2014). Similarly, children as young as 5 have an adultlike understanding of “scene semantics” (i.e., knowing what objects should appear within a particular type of scene, like understanding a shower should be in a bathroom) (Türk et al., 2026) and can successfully extract scene gist information under rapid presentation (Watson et al., 2025). Thus, while the finding of early representation of length/angle information in PPA is consistent with the early development of scene categorization abilities, future work must explicitly test the hypothesis that PPA supports scene categorization early in development (perhaps by asking children to perform a scene categorization task while undergoing fMRI). Additionally, future work can examine how length and angle information, specifically, is used for scene categorization during childhood.

Similarly, although the current study did not explicitly test visually-guided navigation abilities, such abilities depend on spatial information about local scene elements (e.g., the size and shape – that is, the relative lengths and angles – of boundaries and obstacles in service of visually-guided navigation) (Bonner and Epstein, 2017, Dillon et al., 2018, Kamps et al., 2016). Consistent with this adult literature, our finding that OPA robustly represents length/angle information later in development, by age 8, dovetails with a few other behavioral studies suggesting that visually-guided navigation develops late. Obstacle avoidance and efficient path taking – two key features of visually-guided navigation – are slow to develop, not reaching adult-like levels until around 8 years of age (Berard and Vallis, 2006, Choi et al., 2016, Michel et al., 2010, Pryde et al., 1997). More specifically, younger children tend to slow down and take shorter steps as they approach barriers in an obstacle course, whereas the older children and adults will move through the course with a more consistent velocity. Additionally, the ability to remember local boundary information (which depends on OPA in adulthood; Julian et al., 2016) also undergoes protracted development, again reaching maturity around age 8 (Julian et al., 2019). Therefore, while the finding of late developing sensitivity to length/angle information in OPA is consistent with the late development of visually-guided navigation, future work must explicitly test the hypothesis that OPA supports visually-guided navigation late in development (again, perhaps by asking children to perform a navigation task while undergoing fMRI). Future work may also ask how length and angle information is specifically used in visually-guided navigation, or even other functions.

It is remarkable that length and angle infromation is not robustly represented in OPA in young children, despite these children (and even younger children, like preschoolers and toddlers) clearly being able to navigate the immediately visible environment. Along with prior findings that OPA does not represent other types of navigationally-relevant information at age 5 (Jung et al., 2024, Kamps et al., 2020, Rennert and Dilks, 2025), these findings raise the question as to what neural systems support these early visually-guided navigation behaviors. Interestingly, our present results suggest that perhaps even PPA could support this early behavior, given its early developing length and angle sensitivity (although this possibility is not parsimonous with prior literature suggesting that PPA is not involved in visually-guided navigation; Persichetti and Dilks, 2018). Another possibility is that OPA may still support early visually-guided navigation by representing other kinds of navigationally-relevant information. Such representations might also be revealed with more sensitive measures, such as more engaging task manipulations or larger samples. Regardless, the possibility of finding some navigationally-relevant information earlier in OPA does not preclude the hypothesis that OPA undergoes protracted development, since the presence of some navigational information early in development does not imply full functional capacity.

Finally, our findings emphasize the need to use sensitive methods when modeling neurodevelopmental change. In the current study, categorical age-group comparisons suggested that only the oldest participants demonstrated clear length and angle sensitivity in OPA, yet continuous modeling revealed that this sensitivity begins to emerge earlier, before it reaches robust, group-level detectability. This finding is consistent with the Bayesian statistics: the anecdotal evidence in favor of the null hypothesis (i.e. lack of length/angle sensitivity) in either the 5- or 6-year-old OPA reflects initially weak, but gradually strengthening, representations of length and angle information. More broadly, these convergent analyses highlight the value of combining multiple approaches (i.e., categorical and continuous) when characterizing neural development.

In conclusion, this study sought neural evidence that OPA develops later than PPA by using fMRI adaptation to ask when sensitivity to length and angle information develops in scene-selective cortex between the ages of 5 and 8. These findings revealed that such sensitivity emerges earlier in PPA than OPA, aligning neural development with behavioral differences in the development of scene categorization and visually-guided navigation.

## Funding

This work was supported by T32 EY007092 (RJR).

## CRediT authorship contribution statement

**Rebecca J. Rennert:** Writing – review & editing, Writing – original draft, Visualization, Methodology, Investigation, Funding acquisition, Formal analysis, Data curation, Conceptualization. **Daniel D. Dilks:** Writing – review & editing, Supervision, Methodology, Funding acquisition, Conceptualization.

## Declaration of Competing Interest

The authors declare that they have no known competing financial interests or personal relationships that could have appeared to influence the work reported in this paper.

## Data Availability

Data will be made available on request.
