# Supplementary material for: Sensitivity to length and angle information develops early in PPA, but late in OPA
Source: Dev Cogn Neurosci. 2026 Aug 15;81:101800. doi: 10.1016/j.dcn.2026.101800 (PMC13521098; doi:10.1016/j.dcn.2026.101800)
Supplement: Supplementary file 1 [file mmc1.docx]

**Supplemental Materials:**

*Supplemental Figure 1. Early visual cortex (V1) does not even show adaptation.*  Percent signal change (y-axis) to each experimental condition (Different: blue, Length/Angle: yellow, Same: grey) relative to a baseline fixation for 5-, 6-, 7-, and 8-year-old participants. Individual dots represent data from individual participants. Bars represent the mean response to a particular condition for a particular age group (x-axis), and error bars represent SEM.

*Supplemental Figure 2. Both PPA and OPA are scene selective across all age groups*. Percent signal change (y-axis) to each localizer condition (Scenes, Objects) relative to a baseline fixation for PPA (left) and OPA (right) for 5-, 6-, 7-, and 8-year-old participants. Individual dots represent data from individual participants. Bars represent the mean response to a particular condition for a particular age group (x-axis), and error bars represent SEM.

*Supplemental Figure 3. RSC does not represent length and angle information in scenes between 5 and 8 years of age.* Percent signal change (y-axis) to each experimental condition (Different: blue, Length/Angle: yellow, Same: grey) relative to a baseline fixation for 5-, 6-, 7-, and 8-year-old participants. Individual dots represent data from individual participants. Bars represent the mean response to a particular condition for a particular age group (x-axis), and error bars represent SEM.

*How does object selective cortex represent length and angle information in scenes?*

As an exploratory analysis, we asked how object selective cortex (i.e., lateral occipital complex, or LOC) represents changes in length and angle information in scenes throughout development. In adults, object selective cortex (i.e., LO and pFs, which comprise LOC) demonstrates adaptation to scenes (i.e., greater response to Different than Same condition), as well as sensitivity to length/angle changes in scenes (i.e., greater response to Length/Angle than Same condition) (Dillon et al., 2018). So, how does object selective cortex respond to these conditions in childhood?

First, we defined LOC using the GSS method, as described in the main manuscript. Probabilistic maps for LOC were derived from Julian et al. (2012). In the current study, the independent localizer was designed to identify scene-selective regions, and thus only contained scene and object stimuli. Ideally, LOC would be defined by identifying the top 10% of voxels (within the LOC probabilistic map) that respond more to objects than scrambled objects. As we do not have a scrambled object condition, we defined LOC by identifying the top 10% of voxels that respond more to objects than fixation. To validate this alternative approach, we then used a split-half design to look at responses in LOC to scenes and objects. In all ages, LOC responded more to the object than scene condition (5: t_(6)_ = 3.37, p < .01, d = 1.19, 6: t_(8)_ = 4.36, p < .01, d = 1.45, 7: t_(8)_ = 6.63, p < .001, d = 2.21, 8: t_(5)_ = 4.07, p < .01, d = 1.66) (*Supplemental Figure 4).*

We next asked how LOC responded to the three experimental conditions (*Supplemental Figure 5*). Unlike Dillon et al. (2018), we did not even find a significant effect of condition within 5, 6, or 7-year-olds (5: F_(2,22)_ = 0.81, p = .46, ƞ_p_^2^= .07; 6: F_(2,22)_ = 1.04, p = .37, ƞ_p_^2^= .08; 7: F_(2,22)_ = 2.29, p = .13, ƞ_p_^2^= .17). Within the 8-year-olds, however, a 3-way ANOVA revealed a significant effect of condition (F_(2,20)_ = 10.32, p < .001, ƞ_p_^2^= .51). Main effect contrasts revealed the expected adaptation to scenes (greater response to Different than Same condition, t_(10)_ = 3.57, p < .01), but no significant sensitivity to length/angle changes in scenes (no difference between Length & Angle and Same conditions, t_(10)_ = -0.38, p = .72)

We interpret these results as follows. First, perhaps LOC truly does not represent changes in length/angle information in scenes this young in development. This interpretation is consistent with a similar study that found protracted development within LOC using a blocked fMRI adaptation paradigm (Nishimura et al., 2015). Children (ages 5-10), adolescents (ages 11-17), and adults participated in both size and viewpoint adaptation experiments. All three age groups demonstrated size invariance in LOC, but only the adults showed evidence of view invariance in LOC (this effect was weaker in the adolescent group, with the child group not even showing the expected adaptation effect to different objects, like what we find here). Thus, certain object properties develop differentially within LOC, with size invariance developing early, but viewpoint invariance still developing into early adulthood. Perhaps sensitivity to length/angle in scenes is another late-developing feature of LOC.

A second (and not mutually exclusive) possibility is that we may find different results if we were able to separately analyze LO and pFs (i.e., perhaps there is differential development within object-selective regions) and/or define these regions using the more traditional objects > scrambled objects contrast. Future experiments should also ask how object-selective cortex represents length/angle changes in *objects* throughout development. Such an experiment could also investigate if length/angle sensitivity is category-dependent in LOC by asking if geometric properties of scenes and objects develop differentially.

*Supplemental Figure 4. LOC responds more to objects than scenes across all age groups*. Percent signal change (y-axis) to each localizer condition (Objects, Scenes) relative to a baseline fixation for LOC for 5-, 6-, 7-, and 8-year-old participants. Individual dots represent data from individual participants. Bars represent the mean response to a particular condition for a particular age group (x-axis), and error bars represent SEM.

*Supplemental Figure 5. LOC does not represent length and angle information in scenes between 5 and 8 years of age*. Percent signal change (y-axis) to each experimental condition (Different: blue, Length/Angle: yellow, Same: grey) relative to a baseline fixation for 5-, 6-, 7-, and 8-year-old participants. Individual dots represent data from individual participants. Bars represent the mean response to a particular condition for a particular age group (x-axis), and error bars represent SEM.
